# Multiple Chronic Diseases Associated With Tooth Loss Among the US Adult Population

**DOI:** 10.3389/fdata.2022.932618

**Published:** 2022-07-01

**Authors:** Yuqing Zhang, Suzanne G. Leveille, Ling Shi

**Affiliations:** ^1^College of Nursing, University of Cincinnati, Mason, OH, United States; ^2^Robert and Donna Manning College of Nursing and Health Sciences, University of Massachusetts Boston, Boston, MA, United States; ^3^Harvard Medical School, Boston, MA, United States; ^4^Beth Israel Deaconess Medical Center, Boston, MA, United States

**Keywords:** big data, tooth loss, systemic diseases, multimorbidity, public health

## Abstract

**Background:**

Half of US adults aged 20–64 years have lost at least one permanent tooth; one in six adults aged 65 and over in the USA is edentulous. Tooth loss and edentulism interfere with nutritional intake and quality of life. Although selected chronic diseases (e.g., diabetes) have been identified as possible risk factors for tooth loss, data on multiple chronic diseases and on having two or more concurrent chronic diseases (multimorbidity) in relation to tooth loss are lacking. Therefore, this study aimed to assess the association between multiple chronic diseases, multimorbidity, and tooth loss in US adults.

**Methods:**

We performed a secondary data analysis using the US 2012 Behavioral Risk Factor Surveillance System (BRFSS), a national cross-sectional telephone survey studying health conditions and health behaviors among US adults (≥18 years) who are non-institutionalized residents. Variables were derived from the BRFSS Standard Core Questionnaire. Descriptive analysis including means, standard deviations (SDs), and percentages was calculated. Sample weights were applied. The stepwise multinomial logistic regression method was used to examine the relationship between several chronic diseases and tooth loss. Separate multinomial logistic regression models were used to examine the relationship between multimorbidity and tooth loss among all adults aged more than 18 years, adults aged 18–64 years, and adults aged more than 65 years, respectively.

**Results:**

Among the samples (*n* = 471,107, mean age 55 years, 60% female), 55% reported losing no tooth loss, 30% reported losing one to five teeth, 10% reported losing six or more but not all teeth, and 5% reported losing all teeth. After adjusting for demographic characteristics, socioeconomic status, smoking, BMI, and dental care, chronic diseases that were associated with edentulism were chronic obstructive pulmonary disease (COPD) [adjusted risk ratio (adj. RR) 2.18, 95% confidence interval (CI) 2.08–2.29]; diabetes (adj. RR 1.49, 95% CI 1.44–1.56); arthritis (adj. RR 1.49, 95% CI 1.44–1.54); cardiovascular disease (adj. RR 1.38, 95% CI 1.30–1.45); stroke (adj. RR 1.31, 95% CI 1.24–1.40); kidney disease (adj. RR 1.16, 95% CI 1.08–1.25); cancer (adj. RR 1.05, 95% CI 1.01–1.11); and asthma (adj. RR 1.07, 95% CI 1.02–1.12). For those who reported losing six or more teeth, the association remained significant for all the chronic diseases mentioned, albeit the magnitude of association appeared to be comparative or smaller. In addition, adults with multimorbidity were more likely to have tooth loss (loss of one to five teeth: adj. RR 1.17, 95% CI 1.14–1.19; loss of six or more teeth: adj. RR 1.78, 95% CI 1.73–1.82; edentulous: adj. RR 2.03, 95% CI 1.96–2.10).

**Conclusions:**

Multiple chronic diseases were associated with edentulism and tooth loss. People with multimorbidity are more likely to be edentulous than those with one or no chronic disease. The findings from this study will help to identify populations at increased risk for oral problems and nutritional deficits, thus the assessment of oral health should be evaluated further as an important component of chronic illness care.

## Introduction

Tooth loss is a highly prevalent but preventable oral health issue. Half of US adults aged between 20 and 64 years have lost at least one permanent tooth (Dye et al., 2015). One in six elders aged 65 and older in the USA is edentulous (Centers for Disease Control Prevention, 2019). Edentulism is the state of having lost all his/her teeth. It is a severe oral health outcome and is considered an oral handicap (Albrektsson et al., 1987). Tooth loss and edentulism negatively impact people's nutritional intake. People with more tooth loss ingested significantly less dietary fiber, vitamin C, and other nutrients than those without tooth loss (Nowjack-Raymer and Sheiham, 2007). Tooth loss also increases discomfort caused by pain and distress, and negatively impact self-esteem and quality of life (Jones et al., 2003; Emami et al., 2013). Severe tooth loss and edentulism are significant health issues, especially among the elderly. To curb this health problem, Healthy People 2030 has a goal in place to “reduce the proportion of adults aged 45 years and over who have lost all their teeth” (Office of Disease Prevention and Health Promotion).

In recent years, the oral-systemic connection has been largely recognized by the dental and medical professions. Emerging studies were carried out and continue to reveal that oral health impacts systemic health and *vice versa*. As the major cause of tooth loss, periodontal disease was associated with several systemic diseases under the inflammatory mechanism (Kim and Amar, 2006; Linden et al., 2013; Genco and Sanz, 2020). Tooth loss has been associated with chronic diseases and conditions, such as diabetes (Kapp et al., 2007; Winning et al., 2017), cardiovascular disease (Okoro et al., 2005; Winning et al., 2020), stroke (Joshipura et al., 2003), cancer (Maisonneuve et al., 2017; Michaud et al., 2017), obesity (Österberg et al., 2010), depression (Okoro et al., 2012), memory impairment (Okamoto et al., 2015), arthritis (de Pablo et al., 2008), and respiratory diseases such as asthma and chronic obstructive pulmonary disease (COPD) (Wang et al., 2009; Thomas et al., 2010; Bansal et al., 2013; Dwibedi et al., 2020). However, most of these studies have focused on an isolated chronic disease without controlling for other concurrent chronic diseases or multimorbidity. Indeed, due to heterogeneity among these studies in study design, the selection of confounding factors, and the selection of study samples, it is hard to compare the magnitude of the association between tooth loss with each chronic disease across these studies, and to determine the most prominent chronic disease for tooth loss. Therefore, the primary objective of this study was to identify the chronic disease population most at risk for tooth loss using a national representative sample.

Notably, although emerging research evidence has linked tooth loss with several chronic diseases, the relationship between tooth loss and multimorbidity is unclear. Multimorbidity is defined as the coexistence of two or more chronic diseases or conditions in the same individual (World Health Organization, 2016). It has become a major public health concern in recent years as the prevalence of multimorbidity in the USA has increased dramatically with the rise in obesity and population aging (World Health Organization, 2016; Boersma et al., 2020). According to the Centers for Disease Control and Prevention (CDC), 4 in 10 adults in the USA have multimorbidity (Centers for Disease Control Prevention, 2022), one of the leading causes of functional impairment, disability, and mortality, especially among the elderly (Friedman and Shorey, 2019). As people age, the systemic inflammatory burden increases; the inflammatory mechanism is hypothesized to contribute to multimorbidity among older adults, but the reverse may also be true as chronic diseases also contribute to inflammation (Howcroft et al., 2013; Franceschi and Campisi, 2014; Friedman et al., 2015). It is plausible that tooth loss, the distal outcome of periodontal disease that is an oral inflammatory disease, may be associated with multimorbidity. Thus, the second objective of this study is to examine the association between the presence of multimorbidity and tooth loss among the US adult population.

## Materials and Methods

### Study Design and Data Source

We conducted a secondary analysis on data from the US 2012 Behavioral Risk Factor Surveillance System (BRFSS). The choice of the data set was based on the availability of the variable in the survey and the availability of the data set at the time the study was conducted. BRFSS is an annual national cross-sectional telephone survey that collects self-reported data on health conditions and health behaviors among non-institutionalized US adult residents. The telephone interview is conducted by each state health department using the standard questionnaire with technical and methodological assistance from the CDC (Centers for Disease Control Prevention, 2013). The 2012 BRFSS questionnaire contains 18 sections with 85 questions in the core section. The variables used in this analysis came from the Standard Core Questionnaire. Disproportionate stratified sampling method was used for the BRFSS data (Centers for Disease Control Prevention, 2013). The data set used in this analysis contains 475,687 observations and 120 variables (Centers for Disease Control Prevention, 2019). We included people aged 18 years and older and excluded people who have missing data on tooth loss.

### Key Measurements

Tooth loss was measured by asking participants the following question, “*How many of your permanent teeth have been removed because of tooth day or gum disease? Include teeth lost for infection, but do NOT include teeth lost for other reasons, such as injury or orthodontics?*” According to the choice options available in the survey response, participants were grouped into one of four categories of tooth loss: zero tooth loss, one to five tooth loss, six or more tooth loss, and edentulous (lost all the teeth; Centers for Disease Control Prevention, 2021).

Self-reported diagnoses of eight major chronic diseases from the BRFSS core questionnaire were included in this analysis: diabetes, heart disease, stroke, arthritis, cancer, COPD, kidney disease, and asthma. Eight dichotomous chronic diseases variables (Yes/No) were constructed according to the participant's response to the question, “*Has a doctor, nurse, or other health professional ever told you that you had [name and description of the disease]?.”* Multimorbidity was a dichotomous variable that was defined as having at least two of the eight chronic diseases (Centers for Disease Control Prevention, 2021).

### Other Variables

Confounding factors, such as sociodemographic characteristics, behavioral risk factors, and healthcare factors, included age, gender, race/ethnicity, education, income, smoking status, body mass index (BMI), and dental care use. Age and BMI were continuous variables. Gender was defined as male or female according to data derived from the BRFSS demographic questionnaire. Race/ethnicity was defined based on the participant's combined responses to the three questions “Are you Hispanic or Latino?,” “which one or more of the following would you say is your race?,” and “Which one of these groups would you say best represents your race?.” Participants were grouped into one of six categories: White, Black, Hispanic, Asian, Native Hawaiian or Pacific Islander, and American Indian. For those who reported multi-race in the second question, their race/ethnicity was determined based on their reported “best represented” race category in the third question. Based on whether the person had attended or graduated from college or technical school, they were dichotomized into the yes or no college education category. Smoker (Yes/No) was defined according to whether the participant had smoked at least 100 cigarettes in his/her entire life. Dental care utilization was defined according to the response to the question “*How long has it been since you last visited a dentist or a dental clinic for any reason? Include visits to dental specialists, such as orthodontists*.” People who chose the answer, “*Within the past year*” were classified as having regular dental care; people who answered, “*Within the past 2 years” or “Within the past 5 years”* were classified as having some dental care; people who answered, “*Never” or “5 or more years”* were classified as rarely having dental care.

### Statistical Analysis

Descriptive analyses, including means, standard deviations (SDs), and proportions, were used to describe the sociodemographic characteristics of the sample. BRFFS employed the disproportionate sampling of the rarest groups in a hierarchical sampling method. Therefore, the construct of weights was used in this analysis. BRFSS sample weights were applied to generate disease prevalence among the US population. In BRFSS, the final weight construct was the production of the design weight and raking adjustment, where the design weight is the ratio of adults per phone in the household multiplied by the stratum ratio and the raking adjustment is an iterative process to adjust for disproportionate sampling (Centers for Disease Control Prevention, 2013). Bivariate analysis using Chi-squared testing was used to examine the unadjusted association between tooth loss and each chronic disease and multimorbidity. Multinomial logistic regression models were constructed to examine the adjusted association between chronic diseases and tooth loss, as well as the association between multimorbidity and tooth loss, respectively. Model diagnostics for multicollinearity were performed by examining variance inflation factors (VIF). Sensitivity analysis was performed to examine the model improvement by multiple imputations for missing data. Two-sided hypothesis tests with *p* < 0.01, instead of *p* < 0.05 as the statistically significant level, were used to provide a more parsimonious assessment to offset for the large sample size effect in this population study. Statistical analysis was carried out with STATA version 14 (StataCorp LLC, College Station, TX, USA).

### Ethics

Behavioral Risk Factor Surveillance System data are publicly available and de-identified. Therefore, this work was exempt from review by the institutional review board of the University of Massachusetts Boston.

## Results

We enrolled 471,107 people who met the inclusion and exclusion criteria. The mean age of our sample was 55 years, and 60% were female. The weighted percentages of White, Black, Hispanics, Asian American, Hawaiian/Pacific Islander, American Indian, and Hispanics were 65.8, 12.0, 16.2, 4.6, 0.3, and 1.2%, respectively. More than half of the participants had college education and 22% were from low-income families (annual family income < $250,000) (Table 1).

**Table 1:** Sociodemographic characteristics of the sample (unweighted) and the population (weighted), US adults, Behavioral Risk Factor Surveillance System (BRFSS), 2012.

	**Unweighted sample (*n* = 471,107)**	**Weighted population (*n* = 241,366,497)**
Age (mean)	55.1	46.8
Female	59.69%	51.46%
Race
White	79.04%	65.78%
Black	8.87%	11.94%
Hispanic	7.69%	16.23%
Asian American	2.17%	4.57%
Hawaiian and Pacific Islander	0.59%	0.33%
American Indian	1.65%	1.15%
College Education	61.62%	56.17%
Low-income (< $25,000)	20.96%	22.16%

**Figure 1:**
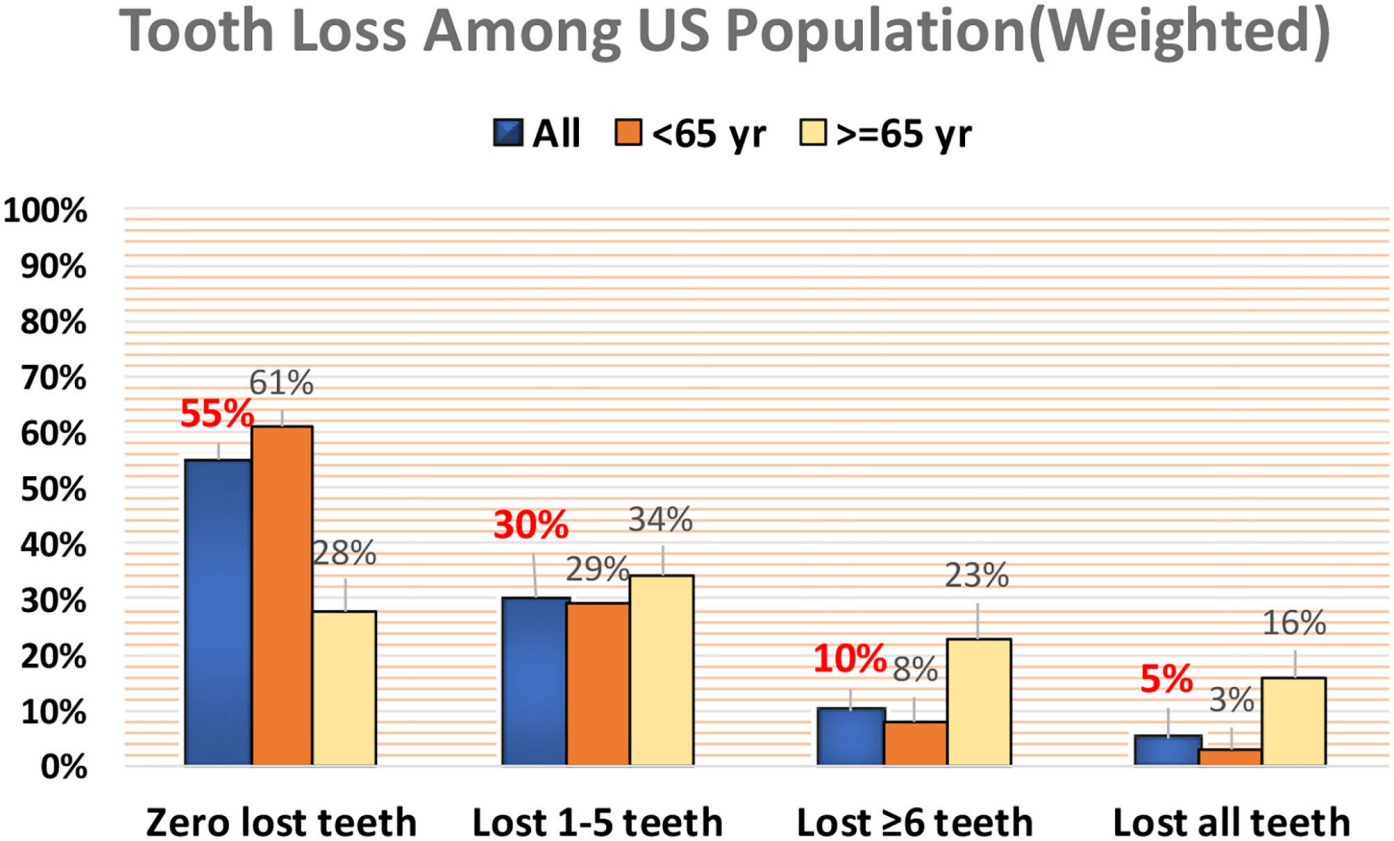
Weighted prevalence of tooth loss among US adults.

**Figure 2:**
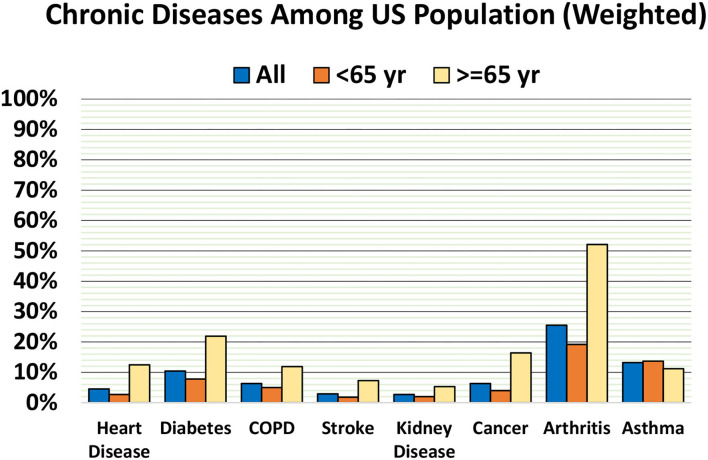
Weighted prevalence of chronic diseases among US adults.

Among the sample, 55% reported losing no teeth, 30% reported losing one to five teeth, 10% reported losing six or more but not all teeth, and 5% reported losing all teeth. The weighted prevalence of tooth loss was much higher among people aged 65 years than those aged below 65 years. The prevalence of lost six or more teeth (but not all teeth) among people aged 65 years and older was 23%, ~3 times higher than people younger than 65 years (8%). The prevalence of edentulism (loss of all teeth) was 16% among people aged 65 years and over, which was more than five times higher than the prevalence of edentulism among people younger than 65 years (Figure 1). Among the chronic diseases included, except for asthma, the weighted prevalence of diabetes, heart disease, COPD, stroke, arthritis, cancer, and kidney disease were higher among people aged 65 years and older than those younger than 65 years (Figure 2).

Demographic characteristics and health risk factors by tooth loss categories are displayed in Table 2. Mean ages increased across groups with greater tooth loss. People who attended college reported less tooth loss than people who did not attend college. Compared to people with higher incomes, those with low incomes were two times as likely to report any tooth loss (67 and 33%, respectively). Smokers were less likely to report no tooth loss than non-smokers (58 vs. 37%), and they were three times more likely to be edentulous than non-smokers (12 vs. 4%). More than half of people who had regular dental care reported no tooth loss, while only 32% of those who sometimes had dental care had no tooth loss. The lowest mean BMI was among people who had no tooth loss, whereas the highest mean BMI was among people who had lost six or more teeth.

**Table 2:** Sociodemographic and health risk factors by tooth loss categories, US adults, Behavioral Risk Factor Surveillance System, 2012.

	**Zero tooth loss** ***N*** **(%)**		**1–5 tooth loss** ***N*** **(%)**		**≥6, not all** ***N*** **(%)**		**Edentulous** ***N*** **(%)**	
Age (mean, std)	48.6	(17.5)	57.5	(15.8)	64.8	(13.4)	69.1	(12.1)
Female	134,788	(47.6)	86,036	(30.4)	38,970	(13.8)	23,502	(8.3)
College Education	165,122	(56.7)	86,595	(29.7)	28,626	(9.8)	11,130	(3.8)
**Race**
White	181,541	(49.3)	109,368	(29.7)	47,990	(13.0)	29,053	(7.9)
Black	14,527	(35.2)	14,095	(34.2)	8,524	(20.7)	4,108	(9.9)
Hispanic	17,557	(48.9)	12,754	(35.6)	3,858	(10.8)	1,672	(4.7)
Asian	6,232	(61.6)	2,998	(29.6)	699	(6.9)	192	(1.9)
Hawaiian	1,342	(49.0)	1,043	(38.1)	266	(9.7)	86	(3.1)
Indian	2,915	(38.1)	2,498	(32.6)	1,433	(18.7)	814	(10.6)
**Income**
<10,000	8,317	(33.0)	7,198	(28.6)	5,660	(22.5)	4,016	(16.0)
10,000–15,000	7,536	(28.6)	7,595	(23.8)	6,410	(24.3)	4,811	(18.3)
15,000–20,000	11,188	(32.9)	10,519	(30.9)	7,087	(20.8)	5,205	(15.3)
20,000–25,000	14,530	(36.0)	13,348	(33.1)	7,689	(19.1)	4,759	(11.8)
25,000–35,000	18,553	(39.2)	16,321	(34.5)	8,153	(17.2)	4,252	(9.0)
35,000–50,000	27,570	(46.1)	20,781	(34.8)	8,170	(13.7)	3,285	(5.5)
50,000–75,000	35,101	(54.9)	20,730	(32.4)	6,053	(9.5)	2,017	(3.2)
>75,000	74,710	(67.0)	29,653	(26.6)	5,700	(5.1)	1,524	(1.4)
**Smoker**
No	145,694	(56.9)	75,238	(29.4)	23,587	(9.2)	11,415	(4.46)
Yes	79,377	(37.5)	68,166	(32.2)	39,410	(18.6)	24,539	(11.6)
**Dental Care**
Regular	169,846	(53.1)	104,634	(32.7)	37,459	(11.7)	7,927	(2.5)
Some	18,735	(32.1)	11,019	(18.9)	9,713	(16.66)	18,830	(32.3)
Rare	38,276	(41.5)	28,989	(31.4)	16,124	(17.5)	8,868	(9.6)
BMI	26.8	(5.4)	27.9	(5.6)	28.4	(6.0)	27.9	(6.0)

Multinomial logistic regression analysis results indicated, after adjusting for demographic characteristics, socioeconomic status, smoking, BMI, and dental care, the chronic diseases that were associated with edentulism were: COPD [adjusted risk ratio (adj. RR) 2.18, 95% confidence interval (CI) 2.08–2.29]; diabetes (adj. RR 1.49, 95% CI 1.44–1.56); arthritis (adj. RR 1.49, 95% CI 1.44–1.54); cardiovascular disease (adj. RR 1.38, 95% CI 1.30–1.45); stroke (adj. RR 1.31, 95% CI 1.24–1.40); kidney disease (adj. RR 1.16, 95% CI 1.08–1.25); cancer (adj. RR 1.05, 95% CI 1.01–1.11); and asthma (adj. RR 1.07, 95% CI 1.02–1.12) (Table 3). For those who reported having lost six or more teeth, the associations remained significant for all the above mentioned chronic diseases, albeit the magnitude of associations was comparable or smaller. A clear dose-response pattern was observed between the magnitudes of the association with chronic diseases and the severity of tooth loss. For example, compared to people without COPD, those with COPD were 14% more likely to have lost one to five teeth, 81% more likely to have lost six or more teeth, and more than two times more likely to be edentulous. The risks of tooth loss varied for different chronic diseases. COPD was found to have the strongest association with tooth loss. Moderate associations were observed among people with diabetes, arthritis, heart disease, or stroke. Weak associations with tooth loss were observed among people with kidney disease, cancer, or asthma (Table 3).

**Table 3:** Factors associated with tooth loss, US adults, BRFSS, 2012.

**Independent variables**	**Dependent variable: Number of tooth loss^a^**			
	**Zero**	**1–5 teeth**	**≥6 but not all teeth**	**Edentulous**
		**Adj. RR (95% CI)**	**Adj. RR (95% CI)**	**Adj. RR (95% CI)**
**Sociodemographic characteristics**
Age	1	1.03 (1.03, 1.04)	1.06 (1.06, 1.06)	1.09 (1.09, 1.09)
Female	1	0.95 (0.93, 0.96)	0.95 (0.93, 0.98)	1.14 (1.11, 1.18)
Race				
White	1	1	1	1
Black	1	1.88 (1.82, 1.93)	2.59 (2.50, 2.70)	1.88 (1.78, 1.98)
Hispanic	1	1.47 (1.43, 1.51)	1.07 (1.02, 1.12)	0.68 (0.63, 0.73)
Asian American	1	1.45 (1.37, 1.53)	1.31 (1.19, 1.45)	0.74 (0.62, 0.89)
Hawaiian and PI	1	1.72 (1.57, 1.90)	1.20 (1.03, 1.43)	0.62 (0.48, 0.83)
American Indian	1	1.48 (1.39, 1.58)	1.82 (1.68, 1.98)	1.71(1.53, 1.90)
College Education	1	0.67 (0.65,0.68)	0.49 (0.48, 0.51)	0.36 (0.35, 0.37)
Low-income	1	1.18 (1.15, 1.20)	2.03 (1.98, 2.09)	2.23 (2.15, 2.31)
**Chronic diseases**
COPD	1	1.14 (1.10, 1.19)	1.81 (1.74,1.88)	2.18 (2.08, 2.29)
Diabetes	1	1.05 (1.02, 1.08)	1.35 (1.31, 1.39)	1.49 (1.44, 1.56)
Arthritis	1	1.17 (1.14, 1.19)	1.51 (1.48, 1.55)	1.49 (1.44, 1.54)
Heart Disease	1	1.11 (1.07, 1.15)	1.29 (1.25, 1.36)	1.38 (1.31, 1.45)
Stroke	1	0.99 (0.95, 1.05)	1.14 (1.08, 1.21)	1.32 (1.24, 1.40)
Kidney Disease	1	1.08 (1.03, 1.13)	1.17 (1.10, 1.24)	1.16 (1.08, 1.25)
Cancer	1	1.02 (0.99, 1.05)	1.09 (1.05, 1.13)	1.05 (1.01, 1.10)
Asthma	1	0.99 (0.97, 1.02)	1.08 (1.04, 1.11)	1.07 (1.02, 1.12)
**Health risk factors**				
Dental care				
Regular	1	1	1	1
Some	1	0.69 (0.67, 0.71)	1.19 (1.14, 1.23)	110.88 (10.42, 11.35)
Rare	1	1.13 (1.11, 1.16)	1.57 (1.52, 1.61)	4.44 (4.26, 4.63)
Smoker	1	1.56 (1.54, 1.59)	2.82 (2.75, 2.89)	3.78 (3.66, 3.92)
BMI	1	1.03 (1.03, 1.03)	1.04 (1.03, 1.04)	1.02 (1.01, 1.02)

*COPD, chronic obstructive pulmonary disease; BMI, body mass index; AOR, adjusted odds ratio; CI, confidence interval*.

a *Multinomial logistic regression model, adjusted for sociodemographic factors (age, sex, education, income, and race), health behavioral factors (smoking status, body mass index, and dental care), and chronic diseases*.

Having two or more concurrent chronic diseases (multimorbidity) was associated with a higher risk of tooth loss compared to those with no or only one chronic disease. Specifically, people with multimorbidity were 17% more likely to lose one to five teeth, 78% more likely to lose six or more teeth, and more than two times as likely to be edentulous than people without multimorbidity (Table 4).

**Table 4:** Association between multimorbidity and tooth loss by age group^a^.

	**Zero tooth loss (Reference)**	**Lost 1–5 teeth Adj. RR (95% CI)**	**Lost ≥6 teeth Adj. RR (95% CI)**	**Edentulous Adj. RR (95% CI)**
Multimorbidity (all ages)	1	1.22 (1.20, 1.25)	1.90 (1.86, 1.96)	2.15 (2.07, 2.22)
Multimorbidity (18–64 years)	1	1.29 (1.25, 1.33)	2.26 (2.18, 2.35)	2.86 (2.71, 3.01)
Multimorbidity (≥65 years)	1	1.25 (1.11, 1.19)	1.56 (1.50, 1.61)	1.66 (1.59, 1.74)

a *Multinomial logistic regression models, adjusted for sociodemographic factors (age, sex, education, income, and race) and health behavioral factors (smoking status, body mass index, and dental care)*.

Missing data distribution was examined for the dependent and independent variables in the multinomial logistic regression model. Income was the variable with the most missing data, with 66,745 missing accounting for 14% of the total sample. Following in order are the diabetes status, race group, and smoking status, with about 1.6–2.5% missing data. Multiple imputations were performed for all variables in the model. After imputation, the model was run again and the *R*^2^ after imputation was 0.175, which indicates almost no change to the previous *R*^2^ (0.178). In addition, the imputation made no material change to the coefficients and significance of the previous analyses. Therefore, the model was deemed to be insensitive to imputation, so the reported results were based on the data without imputation. Model diagnostic for multicollinearity was performed. Multicollinearity of included predictors was refuted by checking the VIF. The VIF equals 1.18, which is <10, indicating that multicollinearity is not a concern in this model.

## Discussion

This study filled a gap in previous research to understand the association between several factors, including demographic characteristics, socioeconomic status, health behaviors, chronic diseases, and multimorbidity, with tooth loss. Using data derived from a large population-based study, we included eight common and major chronic diseases in our analysis, thus allowing us to examine and compare the associations between them with tooth loss in the same context.

The prevalence of tooth loss reported in this study is generally consistent with a previous national study (Dye et al., 2015). Dye et al. (2015) examined the National Health and Nutrition Examination Survey (NHANES) 2011–2012 data and reported that about 52% of adults aged 20–64 years in the USA have some extent of tooth loss; 19% of people aged 65 and older are edentulous. We found a slightly lower prevalence of tooth loss in the BRFSS sample. One of the possible explanations for the slightly lower percentages of tooth loss in our study is that in BRFSS, participants were asked about tooth loss due to cavity and periodontal diseases, excluding tooth loss due to injuries, orthodontics, and prosthetic reasons. Therefore, the prevalence statistics reported in this study provided a more parsimonious outcome measure for tooth loss for our hypothesis.

Edentulism is a severe oral disability among older adults. Previous longitudinal studies have shown that advanced age is associated with an increased risk of periodontal disease (Burt et al., 1990). As people age, the functionality of the osteoblasts cell in periodontal tissues decreases and alveolar bone loss contributes to tooth mobility (Huttner et al., 2009). In addition, gingival recession is commonly seen among older people and is related to increased root caries and tooth loss among seniors (Gati and Vieira, 2011; AlQranei et al., 2021). In this study, we found that the edentulous prevalence among people aged 65 and over is five times higher than that of people under 65 years. Tooth loss is highly associated with aging. In this study, each 1-year increase in age is associated with a 9% increase in the likelihood of being edentulous after controlling for all other factors in the model. After controlling for other risk factors, this is translated to about 2.5 times increased risk of being edentulous for every 10 years of aging.

In recent decades, increased focus has been put on the association between tooth loss and commonly seen chronic diseases, such as diabetes, heart disease, respiratory diseases, and cancer (Gill et al., 2020). The inclusion of eight commonly seen chronic diseases in our analysis allowed us to compare and rank them according to their degree of association with tooth loss. We found four chronic diseases, heart disease, diabetes, arthritis, and COPD, to be strongly associated with tooth loss. Alternatively, cancer or asthma had the weakest associations with tooth loss, not a clinically meaningful finding. Our findings on the strong association between tooth loss and diabetes, heart disease, arthritis, and COPD are consistent with the conclusions of previous studies (Wang et al., 2009; Demmer et al., 2011; Muñoz-Torres et al., 2017; Winning et al., 2017, 2020; Cheng et al., 2018; Kim et al., 2019; Albrecht et al., 2021). Additionally, we highlighted that the tooth loss risk caused by COPD might be higher than other chronic diseases. People with COPD may experience a more significant burden of persistent inflammation over the years than people with other chronic diseases, including asthma. Asthma may contribute less significantly to systemic inflammation due to the short onset of episodes of acute asthma attacks. Previous studies indicated that COPD significantly increases the systemic inflammatory burden, which could be further amplified by smoking (Qian et al., 2020; Andreeva et al., 2021). Smoking is a well-known risk factor for COPD and heart disease, as well as tooth loss (Cunningham et al., 2016). A systematic review based on cross-sectional studies and cohort studies reported that the increased odds of tooth loss among smokers range from 1.69 to 4.04 (Hanioka et al., 2011), comparable to our findings. The magnitude of association is substantial, but is consistent with a previous study (Hanioka et al., 2011). The combined effect of smoking and COPD on tooth loss is complex and not well-understood. Therefore, further investigation in longitudinal studies is warranted.

We found that multimorbidity is strongly associated with tooth loss. Using a process mining technique, Larvin et al. (2021) suggested that tooth loss might contribute to the developmental trajectories of multimorbid diseases. However, to the best of our knowledge, only one study has examined the association between multimorbidity and tooth loss in a large-scale population study; using data from the Brazilian national health survey, Bomfim et al. (2021) reported older adults (≥60 years) with multimorbidity were 17% more likely to lose functional dentition (i.e., lost more than 23 teeth or be edentulous) compared to those without multimorbidity. Notably, the association was even stronger among young adults (18–59 years); those with multimorbidity were 32% more likely to lose functional dentition than their counterparts with one or no chronic disease. In our study, the adjusted relative risk ratio of having tooth loss in the presence of multimorbidity was much higher than that observed by Bomfim. However, our findings regarding the differences by age groups were similar to those of the Bomfim study. It is plausible that because tooth loss is associated with aging in general, consistent with the increased inflammatory burden of aging, the effect of having multimorbidity on tooth loss is somewhat attenuated among older adults.

Though much more work is needed to understand the biological pathway underlying the association between multimorbidity and tooth loss, research evidence suggests that inflammation associated with multimorbidity may play a role. It could be that people with multimorbidity might have a greater inflammatory burden throughout the body, which produces more proinflammatory cytokines that in turn exacerbate periodontium infection, or *vice versa* (Friedman et al., 2015). Another possible explanation might be that people with multimorbidity use polypharmacy defined as taking five or more medications (Masnoon et al., 2017) and this, in turn, may contribute to tooth loss. Polypharmacy contributes to dry mouth, specifically, anticholinergics, opioids, antidepressants, bronchodilators, and some cardiovascular agents can induce dry mouth (Villa et al., 2015). Persistent dry mouth increases the risk of dental caries and oral infections (Thomson et al., 2021). People with multimorbidity are often prescribed multiple medications to treat symptoms and to prevent complications associated with chronic diseases. The risk for dry mouth-related oral problems increases according to total dosages and duration of use (Johanson et al., 2015). Consequently, people with multimorbidity and polypharmacy may lose more teeth than others without multimorbidity. However, further studies are needed to investigate this hypothesis. The last possible explanation might be that both multimorbidity and tooth loss are social determinants of health issues. Therefore, these two conditions might have presented hand-in-hand among people with a disadvantaged socioeconomic status. In this study, college-educated people were less likely to be edentulous and people with low incomes were more likely to lose six or more teeth or be edentulous. Although we adjusted for these factors, there may be other confounding factors that we could not account for here. Our findings are consistent with previous studies in which social determinants of health factors presented a substantial impact on tooth loss (Jiang et al., 2013; Lee et al., 2022). We also found that access to regular dental care was associated with teeth retention; compared to those with regular dental care, people with less dental care were more likely to be edentulous. People with multimorbidity and those with a higher degree of tooth loss may share similar social determinants of health. On one hand, previous observational and longitudinal studies indicated that socioeconomically disadvantaged population was more likely to have an earlier onset and higher prevalence of multimorbidity (Pathirana and Jackson, 2018; Dugravot et al., 2020; Khanolkar et al., 2021). On the other hand, due to a lack of dental insurance and high out-of-pocket payment for dental services, the socioeconomically disadvantaged population often lacks adequate dental care than their affluent counterparts, which could further worsen their oral health and result in more tooth loss over the years.

This study has some strengths and limitations. First, the BRFSS is a cross-sectional study, we cannot confirm the directionality of the association between tooth loss and multiple chronic diseases. The bidirectional association between multiple chronic diseases and tooth loss is plausible, but this warrants further investigation in longitudinal and cohort studies. Second, the data are self-reported in the BRFSS. Self-reported data are susceptible to recall or reporting bias. However, such bias usually contributes to a more conservative result because diagnoses are more likely to be underreported than overreported when the data are self-reported. For example, people could have undiagnosed diabetes. Lastly, we cannot have all the desired variables included in our models using secondary data analysis. For example, oral health behaviors and oral disease diagnoses could influence tooth retention although these variables were not collected in the BRFSS. The strength of this study is that it is based on a large, nationally representative sample making the results generalizable to the US adult population.

## Conclusions

Multiple chronic diseases were associated with edentulism and tooth loss although the magnitude of associations with tooth loss varied. Knowing which chronic disease populations are at greater risk of tooth loss will inform the priority setting in healthcare, research, and public health policymaking. People with COPD, diabetes, heart disease, or arthritis should be given more attention to their oral health and be guided to use oral care resources and self-care strategies early in the course of illness to prevent tooth loss as they age. The association between tooth loss, edentulism, and multimorbidity should be further studied through the lens of social determinants of health.

## Data Availability Statement

Publicly available datasets were analyzed in this study. This data can be found here: Behavioral Risk Factor Surveillance System, Centers for Disease Control and Prevention, United States.

## Ethics Statement

BRFSS data is publicly available and de-identified. Therefore, this work was exempt from review by the institutional review board at the University of Massachusetts Boston. Written informed consent for participation was not required for this study in accordance with the national legislation and the inquisitional requirements.

## Author Contributions

YZ contributed to conception, study design, data acquisition, data analysis and interpretation, and drafted the manuscript. SL contributed to conception, study design, interpretation, and critically revised the manuscript. LS contributed to study design, statistical plan development, interpretation, and critically revised the manuscript. All authors contributed to the article and approved the submitted version.

## Conflict of Interest

The authors declare that the research was conducted in the absence of any commercial or financial relationships that could be construed as a potential conflict of interest.

## Publisher's Note

All claims expressed in this article are solely those of the authors and do not necessarily represent those of their affiliated organizations, or those of the publisher, the editors and the reviewers. Any product that may be evaluated in this article, or claim that may be made by its manufacturer, is not guaranteed or endorsed by the publisher.
